# Supplementary material for: Voices from the front lines: A qualitative study of integration of HIV, tuberculosis, and primary healthcare services in Johannesburg, South Africa
Source: PLoS One. 2020 Oct 8;15(10):e0230849. doi: 10.1371/journal.pone.0230849 (PMC7544124; doi:10.1371/journal.pone.0230849)
Supplement: S1 File — (PDF) [file pone.0230849.s001.pdf]

Today's Date					
d	d	m	m	y	r

Participant ID				
K				

Facility ID

Instructions: (Study staff) Use this form to interview district level key informants. Read the questions aloud. For open responses, write the response in space provided. For Yes/No or questions with options, tick the appropriate box. Do not read aloud text in all caps.

SECTION 1: TITLE, ROLE, ETC.

INTRODUCTION: Thanks for taking the time to speak with me. We'll start off with a few questions about you and your position.

Question	Response
1. Who is your employer?	OPEN RESPONSE:
2. How long have you worked for [EMPLOYER]?	__________months __________years
3. What is your official title? (Note that your title will not be presented as part of the results of the study.)	OPEN RESPONSE:
4. a. Has your position/title changed since you've been working for [EMPLOYER]?<br><br>b. [IF YES] How and when?	☐ Yes (1)<br>☐ No (0)<br><br>OPEN RESPONSE:

Today's Date					
d	d	m	m	y	r

Participant ID				
**K**				

<u>SECTION 2: SERVICES AND REFERRALS IN THE DISTRICT</u>

INTRODUCTION: Now I'd like to ask some questions about how health services are provided in the district. I'm most interested in HIV, TB, sexual and reproductive health and non-communicable diseases like cancer, cardiovascular disease, respiratory disease and diabetes. Some of the questions will focus on those services, and some of the questions are more general.

5. First, I'd like to know how many public health facilities there are in this district. Can you tell me how many [SEE COLUMN A BELOW] there are and the names of them?	COMPLETE COLUMNS B AND C IN THE TABLE BELOW.
6. For each facility, can you tell me if it falls under the authority of the city, district or province?	COMPLETE COLUMN D IN THE TABLE BELOW.
7. Then I'd like to know whether certain services are **required** to be offered at each facility.<br><br>For each facility, can you tell me if provision of [LIST SERVICES IN COLUMNS E-N] is required at that facility?	COMPLETE COLUMNs E-N IN THE TABLE BELOW.

Today's Date					
d	d	m	m	y	r

Participant ID				
K				

Table 1 (NB: Tx= treatment, Sc = screening, mgmt. = management)

A. Facility type/ level	B. # of facilities	C. Name of facility(ies)	D. Authority (City, district or province?)	E.HIV/AIDS	F. Tuberculosis	G. Women's Health	H. Reproductive Health	I. Chronic Disease Mgmt	J. Immunisations/ Childhood Health	K. Mental Health	L. Prevention	M. Other
Tertiary hospitals		1.	1.	Y / N	Y / N	Y / N	Y / N	Y / N	Y / N	Y / N	Y / N	Y / N
		2.	2.	Y / N	Y / N	Y / N	Y / N	Y / N	Y / N	Y / N	Y / N	Y / N
		3.	3.	Y / N	Y / N	Y / N	Y / N	Y / N	Y / N	Y / N	Y / N	Y / N
		4.	4.	Y / N	Y / N	Y / N	Y / N	Y / N	Y / N	Y / N	Y / N	Y / N
		5.	5.	Y / N	Y / N	Y / N	Y / N	Y / N	Y / N	Y / N	Y / N	Y / N
Secondary hospitals		1.	1.	Y / N	Y / N	Y / N	Y / N	Y / N	Y / N	Y / N	Y / N	Y / N
		2.	2.	Y / N	Y / N	Y / N	Y / N	Y / N	Y / N	Y / N	Y / N	Y / N
		3.	3.	Y / N	Y / N	Y / N	Y / N	Y / N	Y / N	Y / N	Y / N	Y / N
		4.	4.	Y / N	Y / N	Y / N	Y / N	Y / N	Y / N	Y / N	Y / N	Y / N
		5.	5.	Y / N	Y / N	Y / N	Y / N	Y / N	Y / N	Y / N	Y / N	Y / N
Community health centres or "day hospitals"		1.	1.	Y / N	Y / N	Y / N	Y / N	Y / N	Y / N	Y / N	Y / N	Y / N
		2.	2.	Y / N	Y / N	Y / N	Y / N	Y / N	Y / N	Y / N	Y / N	Y / N
		3.	3.	Y / N	Y / N	Y / N	Y / N	Y / N	Y / N	Y / N	Y / N	Y / N
		4.	4.	Y / N	Y / N	Y / N	Y / N	Y / N	Y / N	Y / N	Y / N	Y / N

Today's Date					
d	d	m	m	y	r

Participant ID				
K				

A. Facility type/ level	B. # of facilities	C. Name of facility(ies)	D. Authority (City, district or province?)	E.HIV/AIDS	F. Tuberculosis	G. Women's Health	H. Reproductive Health	I. Chronic Disease Mgmt	J. Immunisations/ Childhood Health	K. Mental Health	L. Prevention	M. Other
		5.	5.	Y / N	Y / N	Y / N	Y / N	Y / N	Y / N	Y / N	Y / N	Y / N
Primary health clinics		1.	1.	Y / N	Y / N	Y / N	Y / N	Y / N	Y / N	Y / N	Y / N	Y / N
		2.	2.	Y / N	Y / N	Y / N	Y / N	Y / N	Y / N	Y / N	Y / N	Y / N
		3.	3.	Y / N	Y / N	Y / N	Y / N	Y / N	Y / N	Y / N	Y / N	Y / N
		4.	4.	Y / N	Y / N	Y / N	Y / N	Y / N	Y / N	Y / N	Y / N	Y / N
		5.	5.	Y / N	Y / N	Y / N	Y / N	Y / N	Y / N	Y / N	Y / N	Y / N
Mobile health facilities		1.	1.	Y / N	Y / N	Y / N	Y / N	Y / N	Y / N	Y / N	Y / N	Y / N
		2.	2.	Y / N	Y / N	Y / N	Y / N	Y / N	Y / N	Y / N	Y / N	Y / N
		3.	3.	Y / N	Y / N	Y / N	Y / N	Y / N	Y / N	Y / N	Y / N	Y / N
		4.	4.	Y / N	Y / N	Y / N	Y / N	Y / N	Y / N	Y / N	Y / N	Y / N
		5.	5.	Y / N	Y / N	Y / N	Y / N	Y / N	Y / N	Y / N	Y / N	Y / N
Pharmacy depots		1.	1.	Y / N	Y / N	Y / N	Y / N	Y / N	Y / N	Y / N	Y / N	Y / N
		2.	2.	Y / N	Y / N	Y / N	Y / N	Y / N	Y / N	Y / N	Y / N	Y / N
		3.	3.	Y / N	Y / N	Y / N	Y / N	Y / N	Y / N	Y / N	Y / N	Y / N

Today's Date					
d	d	m	m	y	r

Participant ID				
K				

A. Facility type/ level	B. # of facilities	C. Name of facility(ies)	D. Authority (City, district or province?)	E.HIV/AIDS	F. Tuberculosis	G. Women's Health	H. Reproductive Health	I. Chronic Disease Mgmt	J. Immunisations/ Childhood Health	K. Mental Health	L. Prevention	M. Other
		4.<br>5.	4.<br>5.	Y / N<br>Y / N	Y / N<br>Y / N	Y / N<br>Y / N	Y / N<br>Y / N	Y / N<br>Y / N	Y / N<br>Y / N	Y / N<br>Y / N	Y / N<br>Y / N	Y / N<br>Y / N
Other public health facilities (LIST):<br>1.<br>2.<br>3.<br>4.<br>5.		1.<br>2.<br>3.<br>4.<br>5.	1.<br>2.<br>3.<br>4.<br>5.	Y / N<br>Y / N<br>Y / N<br>Y / N<br>Y / N	Y / N<br>Y / N<br>Y / N<br>Y / N<br>Y / N	Y / N<br>Y / N<br>Y / N<br>Y / N<br>Y / N	Y / N<br>Y / N<br>Y / N<br>Y / N<br>Y / N	Y / N<br>Y / N<br>Y / N<br>Y / N<br>Y / N	Y / N<br>Y / N<br>Y / N<br>Y / N<br>Y / N	Y / N<br>Y / N<br>Y / N<br>Y / N<br>Y / N	Y / N<br>Y / N<br>Y / N<br>Y / N<br>Y / N	Y / N<br>Y / N<br>Y / N<br>Y / N<br>Y / N

Today's Date					
d	d	m	m	y	r

Participant ID				
K				

8. a. Are there any NGOs, religious groups or other non-government organizations providing health services in this district?<br><br>b. IF YES, do they provide:<br><br>i. HIV/AIDS Services<br>ii. Tuberculosis Services<br>iii. Women's Health Services<br>iv. Reproductive Health Services<br>v. Chronic Disease Mgmnt<br>vi. Immunisations / Childhood Health<br>vii. Mental Health<br>viii. Prevention Services<br>ix. Other: ________________________	☐ No (0)<br>☐ Yes (1)<br><br>i. ☐ No (0) / ☐ Yes (1)<br>ii. ☐ No (0) / ☐ Yes (1)<br>iii. ☐ No (0) / ☐ Yes (1)<br>iv. ☐ No (0) / ☐ Yes (1)<br>v. ☐ No (0) / ☐ Yes (1)<br>vi. ☐ No (0) / ☐ Yes (1)<br>vii. ☐ No (0) / ☐ Yes (1)<br>viii. ☐ No (0) / ☐ Yes (1)<br>ix. ☐ No (0) / ☐ Yes (1)
9. Now I'd like to know about the general referral patterns for services in the district for primary health care facilities and higher level facilities. Can you tell me...<br><br>a. What secondary hospitals can refer to [TERTIARY HOSPITAL NAME 1, 2, 3, 4, 5]?<br><br>b. What CHCs or day hospitals can refer to [SECONDARY HOSPITAL NAME 1, 2, 3, 4, 5]?<br><br>c. What PHCs can refer to [CHC/DAY HOSPITAL NAME 1, 2, 3, 4, 5]?	FOR EACH GRAY SHADED ROW IN TABLE 2, FILL IN FACILITIES LISTED IN COLUMN C IN TABLE 1.<br><br>FOR EACH FACILITY, LIST FACILITIES THAT REFER TO IT IN THE APPROPRIATE ROW BELOW.

Today's Date					
d	d	m	m	y	r

Participant ID				
K				

Table 2

Tertiary hospitals → *(Copy from column C above)*	1.	2.	3.	4.	5.
9a. Feeder facilities *(List secondary hospitals only)*	1.<br>2.<br>3.<br>4.<br>5.	1.<br>2.<br>3.<br>4.<br>5.	1.<br>2.<br>3.<br>4.<br>5.	1.<br>2.<br>3.<br>4.<br>5.	1.<br>2.<br>3.<br>4.<br>5.
Secondary hospitals → *(Copy from column C above)*	1.	2.	3.	4.	5.
9b. Feeder facilities *(List CHCs or day hospitals only)*	1.<br>2.<br>3.<br>4.<br>5.	1.<br>2.<br>3.<br>4.<br>5.	1.<br>2.<br>3.<br>4.<br>5.	1.<br>2.<br>3.<br>4.<br>5.	1.<br>2.<br>3.<br>4.<br>5.
CHCs or "day hospitals" → *(Copy from column C above)*	1.	2.	3.	4.	5.
9c. Feeder facilities *(List PHCs only)*	1.<br>2.<br>3.<br>4.<br>5.	1.<br>2.<br>3.<br>4.<br>5.	1.<br>2.<br>3.<br>4.<br>5.	1.<br>2.<br>3.<br>4.<br>5.	1.<br>2.<br>3.<br>4.<br>5.

Today's Date					
d	d	m	m	y	r

Participant ID				
K				

10. a. Are there any other important referral patterns that are important to know about in the district?<br><br>b. IF YES, Can you describe those? Why are they important?	☐ No (0)<br>☐ Yes (1)<br><br>IF YES, OPEN RESPONSE:
11. Are referral patterns different for...<br><br>a. HIV/AIDS Services?<br><br>11ai. IF YES, In what way?<br><br>b. Tuberculosis Services (PROBE: MDR/XDR or non-drug resistant TB)?<br><br>11bi. IF YES, In what way?<br><br>c. Women's Health (PROBE: TOP, breast cancer, or cervical cancer)?<br><br>11ci. IF YES, In what way?	☐ No (0)<br>☐ Yes (1)<br>IF YES, OPEN RESPONSE:<br><br>☐ No (0)<br>☐ Yes (1)<br>IF YES, OPEN RESPONSE:<br><br>☐ No (0)<br>☐ Yes (1)<br>IF YES, OPEN RESPONSE:

Today's Date					
d	d	m	m	y	r

Participant ID				
K				

d. Reproductive Health (PROBE: injections, pills, Loop (IUCD), or tubal ligation)?<br><br>11di. IF YES, In what way?	☐ No (0)<br>☐ Yes (1)<br>IF YES, OPEN RESPONSE:
e. Chronic Disease Management?<br><br>11ei. IF YES, In what way?	☐ No (0)<br>☐ Yes (1)<br>IF YES, OPEN RESPONSE:
f. Immunisation / Childhood Health?<br><br>11fi. IF YES, In what way?	☐ No (0)<br>☐ Yes (1)<br>IF YES, OPEN RESPONSE:
g. Mental Health?<br><br>11gi. IF YES, In what way?	☐ No (0)<br>☐ Yes (1)<br>IF YES, OPEN RESPONSE:
h. Prevention?<br><br>11hi. IF YES, In what way?	☐ No (0)<br>☐ Yes (1)<br>IF YES, OPEN RESPONSE:
i. Other?<br><br>11ii. IF YES, In what way?	☐ No (0)<br>☐ Yes (1)<br>IF YES, OPEN RESPONSE:

Today's Date					
d	d	m	m	y	r

Participant ID				
K				

12. How do facilities in the district know what services they are required to offer? [PROBE: policies, documents, etc]	OPEN RESPONSE
13. And how do facilities in the district find out about new policies or new services that they must add or changes that must be made to services?	OPEN RESPONSE

Today's Date					
d	d	m	m	y	r

Participant ID				
K				

14. When a new service must be added or service delivery must be changed, whose responsibility is it to determine how that will be done at the facility level? [PROBE: facility manager, service manager, other?]	OPEN RESPONSE
15. How is budgeting for health services managed in the district? [PROBE: Who sets the budgets? Where does responsibility lie? Timing of budget cycles? Reporting/reconciling?]	OPEN RESPONSE

Today's Date					
d	d	m	m	y	r

Participant ID				
K				

16. How do facilities know how much they can or should spend on various services – e.g. HIV, contraception or other sexual and reproductive health services, non-communicable diseases? [PROBE: Are there guidelines or policies?]	OPEN RESPONSE

SECTION 3: INTEGRATION OF HIV AND TB INTO PHC SERVICES

INTRODUCTION: Finally, I'd like to ask a few questions about integration of HIV and TB services into services at the primary health care level.

17. a. Earlier you mentioned that HIV services are offered at some facilities in this district. At the primary health clinics, are HIV services separate or standalone (e.g. NGO managed or a CCMT site) or are they integrated into other services at the site?<br><br>b. Can you explain how they are offered?	☐ Separate (1)<br>☐ Integrated (2)<br>☐ Depends on the site (3)<br><br>OPEN RESPONSE:

Today's Date					
d	d	m	m	y	r

Participant ID				
K				

18. a. Have there been any efforts to integrate HIV testing and treatment into other services at the primary health clinics in the district?	☐ No (0)<br>☐ Yes (1)
b. IF YES, can you describe those? [PROBE: When? Why? Was it successful?]	OPEN RESPONSE:
c. IF NO, can you say why you think that is?	OPEN RESPONSE:

Today's Date					
d	d	m	m	y	r

Participant ID				
K				

19. a. Integration can be done in many ways. For example, it can mean one health care provider offering many different services in the same visit, or one clinic offering many services on the same day. Do you think integration of HIV into other primary health care services is a good idea?<br><br>b. Why do you think that?	☐ No (0)<br>☐ Yes (1)<br>☐ It depends... (3)<br>☐ Unsure (4)<br><br>OPEN RESPONSE:
20. a. Earlier you mentioned that TB services are offered at some facilities in this district. At the primary health clinics, are TB services separate or standalone or are they integrated into other services at the site?<br><br>b. Can you explain how they are offered?	☐ Separate (1)<br>☐ Integrated (2)<br>☐ Depends on the site (3)<br><br>OPEN RESPONSE:

Today's Date					
d	d	m	m	y	r

Participant ID				
K				

21. a. As I noted above, integration can be done in many ways. Do you think integration of TB into other primary health care services is a good idea?<br><br>b. Why do you think that?	☐ No (0)<br>☐ Yes (1)<br>☐ It depends... (3)<br>☐ Unsure (4)<br><br>OPEN RESPONSE:

Today's Date					
d	d	m	m	y	r

Participant ID				
K				

Interview initials and date: ____

Interviewer comments:

Today's Date					
d	d	m	m	y	r

Participant ID				
F				

Facility ID

Instructions: (Study staff) Use this form to interview facility managers at each site. Read the questions aloud. For open responses, write the response in space provided. For Yes/No or questions with options, tick the appropriate box. Do not read aloud text in all caps.

SECTION 1: TITLE, ROLE, ETC.

INTRODUCTION: Thanks for taking the time to speak with me. We'll start off with a few questions about you and your role here.

Question	Response
1. How long have you worked at this facility?	__________months __________years
2. What is your position or title here?	OPEN RESPONSE:
3. a. Has your position/title changed since you've been working here?<br><br>b. [IF YES] How and when?	☐ Yes (1)<br>☐ No (0)<br><br>OPEN RESPONSE:

Today's Date					
d	d	m	m	y	r

Participant ID				
F				

SECTION 2: STAFFING

INTRODUCTION: Now I'd like to ask you about staffing – for example, what kinds of staff and how many you have here.

4. What kinds of staff do you employ at this facility? FOR EACH TYPE OF STAFF TYPE, ASK: Are there different types or levels? How many of each do you have?	COMPLETE TABLE 1.

Table 1

Type of staff:	Employed in service?	If yes, number:
Specialist<br><br>Doctor<br><br>Registrar	Y / N<br><br>Y / N<br><br>Y / N	___________<br><br>___________<br><br>___________
Primary health care nurse<br><br>Nurse midwife<br><br>Registered nurse<br><br>Enrolled nurse<br><br>Other nurse (LIST):	Y / N<br><br>Y / N<br><br>Y / N<br><br>Y / N<br><br>Y / N	___________<br><br>___________<br><br>___________<br><br>___________<br><br>___________
Social worker<br><br>Counselor<br><br>Lay counselor	Y / N<br><br>Y / N<br><br>Y / N	___________<br><br>___________<br><br>___________
Pharmacist<br><br>Pharmacy assistant	Y / N<br><br>Y / N	___________<br><br>___________
Other (LIST):<br><br>Other (LIST):<br><br>Other (LIST):	Y / N<br><br>Y / N<br><br>Y / N	___________<br><br>___________<br><br>___________

Today's Date					
d	d	m	m	y	r

Participant ID				
F				

5. a. Has your staffing changed significantly in the past 5 years?<br><br>b. Why is that?	☐ Yes (1)<br>☐ No (0)<br><br>OPEN RESPONSE:
6. a. Do all staff have training in HIV and/or TB testing and treatment? Or is that only something staff in those departments/services need to have?<br><br>b. Why is that?	☐ Yes – all staff are trained (1)<br>☐ Some staff outside the TB or HIV departments are trained (2)<br>☐ No – no staff outside the TB or HIV departments are trained (0)<br>☐ Don't know (77)<br>☐ N/A (88)<br><br>OPEN RESPONSE:
7. a. Now, thinking about the staff who work in the HIV or TB departments, do they also have training in providing reproductive health services like family planning, antenatal care, TOP or screening for reproductive cancers (e.g. Pap smears, mammograms, etc)?<br><br>b. Why is that?	☐ Yes – all (1)<br>☐ Some (2)<br>☐ No (0)<br>☐ Don't know (77)<br>☐ N/A (88)<br><br>OPEN RESPONSE:

Today's Date					
d	d	m	m	y	r

Participant ID				
F				

8. a. Now, thinking about the staff who work in the HIV or TB departments, do they also have training in providing services for non-communicable diseases like cardiovascular problems, diabetes, respiratory conditions, etc?<br><br>b. Why is that?	☐ Yes – all (1)<br>☐ Some (2)<br>☐ No (0)<br>☐ Don't know (77)<br>☐ N/A (88)<br><br>OPEN RESPONSE:

SECTION 3: SERVICES

INTRODUCTION: I'm interested to know what services are provided by this facility, how patients get services, and referrals to other facilities.

9. For the following services [ASK ABOUT ITEMS IN COLUMN A], can you tell me [ASK QUESTIONS IN COLUMNS B-D]...	COMPLETE TABLE 2

Table 2 (NB: Tx= treatment, Sc = screening, mgmt. = management)

A. Service	B. Days of the week available	C. Hours available	D. If not offered, where are patients referred to for services?
**HIV / AIDS:**			
Testing & Counselling			
ART Initiation			
Routine Treatment (Adults)			
Routine Treatment (Children)			
PMTCT			
Other			
**Tuberculosis:**			
Testing/Diagnosing			
Treatment/Routine Care			
Other			
**Women's Health:**			
TOP			
Cervical cancer screening			
Cervical cancer treatment			
Breast cancer screening			
Breast cancer treatment			

Today's Date					
d	d	m	m	y	r

Participant ID				
F				

OB / GYN			
Other			
**Reproductive Health:**			
Family planning			
Sexually Transmitted Infections			
Other			
**Chronic Disease Mgmt:**			
Obesity			
Diabetes			
Hypertension			
Respiratory Disease			
Cardiovascular Disease			
Other			
**Immunisations / Childhood Health:**			
**Mental Health:**			
**Prevention:**			
Well Child			
Medical Male Circumcision			
Other			
**Other:**			

10. How do facilities in the district know what services they are required to offer? [PROBE: policies, documents, etc]	OPEN RESPONSE

Today's Date					
d	d	m	m	y	r

Participant ID				
**F**				

11. And how do facilities in the district find out about new policies or new services that they must add or changes that must be made to services?	OPEN RESPONSE
12. When a new service must be added or service delivery must be changed, whose responsibility is it to determine how that will be done at the facility level? [PROBE: facility manager, service manager, other?]	OPEN RESPONSE
13. How is budgeting for health services managed in the district? [PROBE: Who sets the budgets? Where does responsibility lie? Timing of budget cycles? Reporting/reconciling?]	OPEN RESPONSE

Today's Date					
d	d	m	m	y	r

Participant ID				
F				

14. And how is budgeting for health services managed within a facility like this? [PROBE: Who sets the budgets? Where does responsibility lie? Timing of budget cycles? Reporting/reconciling?]	OPEN RESPONSE
15. How do facilities know how much they can or should spend on various services – e.g. HIV, contraception or other sexual and reproductive health services, non-communicable diseases? [PROBE: Are there guidelines or policies?]	OPEN RESPONSE
16. a. In this study we're interested in "integration of services"Integration can be done in many ways. For example, it can mean one health care provider offering many different services in the same visit, or one clinic offering many services on the same day. Do you think integration of HIV into other primary health care services is a good idea?	☐ No (0)<br>☐ Yes (1)<br>☐ It depends... (3)<br>☐ Unsure (4)
b. Why do you think that?	OPEN RESPONSE:

Today's Date					
d	d	m	m	y	r

Participant ID				
F				

17. a. Have there been any efforts to integrate HIV testing and treatment into the services at this clinic?	☐ No (0)<br>☐ Yes (1)
b. IF YES, can you describe those? [PROBE: When? Why? Was it successful?]	OPEN RESPONSE:
c. IF NO, can you say why you think that is?	OPEN RESPONSE:
18. a. As I noted above, integration can be done in many ways. Do you think integration of TB into other primary health care services is a good idea?	☐ No (0)<br>☐ Yes (1)<br>☐ It depends... (3)<br>☐ Unsure (4)
b. Why do you think that?	OPEN RESPONSE:

Today's Date					
d	d	m	m	y	r

Participant ID				
F				

19. a. Have there been any efforts to integrate TB testing and treatment into the services at this clinic?	☐ No (0)<br>☐ Yes (1)
b. IF YES, can you describe those?<br>[PROBE: When? Why? Was it successful?]	OPEN RESPONSE:
c. IF NO, can you say why you think that is?	OPEN RESPONSE:

Today's Date					
d	d	m	m	y	r

Participant ID				
F				

SECTION 4: PATIENTS SERVED AND THEIR HEALTH CARE NEEDS

INTRODUCTION: To finish the interview, I have a few more questions about the clients or patients that you see in the services here.

20. a. Do you have a system for tracking how many patients you see in this facility each month?	☐ No (0)<br>☐ Yes (1)
b. IF NO, Why is that?	OPEN RESPONSE
c. IF YES, Can you describe that system for me?	OPEN RESPONSE
21. Can you tell me, on average, how many patients you see in this facility each month? [IF POSSIBLE OBTAIN STATS FOR THE LAST THREE MONTHS.]	Number: ____________ last month<br>Number: ____________ month before last<br>Number: ____________ three months ago<br>Average per month: _____________

Today's Date					
d	d	m	m	y	r

Participant ID				
**F**				

Interviewer initials and date____________________

Interviewer comments:

Today's Date					
d	d	m	m	y	r

Participant ID				
S				

Facility ID

Instructions: (Study staff) Use this form to interview service managers at each site. Read the questions aloud. For open responses, write the response in space provided. For Yes/No or questions with options, tick the appropriate box. Do not read aloud text in all caps.

SECTION 1: TITLE, ROLE, ETC.

INTRODUCTION: Thanks for taking the time to speak with me. We'll start off with a few questions about you and your role here.

Question	Response
1. How long have you worked at this facility?	__________months __________years
2. What is your position or title here?	OPEN RESPONSE:
3. a. Has your position/title changed since you've been working here?<br><br>b. [IF YES] How and when?	☐ Yes (1)<br>☐ No (0)<br><br>OPEN RESPONSE:
4. What service(s) do you oversee at this facility?	☐ HIV/AIDS<br>☐ TB<br>☐ Women's Health<br>☐ Reproductive Health<br>☐ Chronic Disease Mgmt<br>☐ Immunisations/Childhood Health<br>☐ Mental Health<br>☐ Prevention<br>☐ Other: SPECIFY BELOW<br><br>________________________

Today's Date					
d	d	m	m	y	r

Participant ID				
**S**				

SECTION 2: STAFFING

INTRODUCTION: Now I'd like to ask you about staffing – for example, what kinds of staff and how many you have here.

5. Because of your role here, the following questions will be for the:<br><br>IF S/HE IS RESPONSIBLE FOR MORE THAN ONE SERVICE, USE EXTRA COPIES OF THIS PAGE, AND ASK SEPARATELY FOR EACH SERVICE.	LIST SERVICE NAME:<br><br>______________________________
6. What kinds of staff do you employ in this service? FOR EACH TYPE OF STAFF TYPE, ASK: Are there different types or levels? How many of each do you have?	COMPLETE TABLE 1.

Table 1

**Type of staff:**	**Employed in service?**	**If yes, number:**
Specialist	Y / N	___________
Doctor	Y / N	___________
Registrar	Y / N	___________
Primary health care nurse	Y / N	___________
Nurse midwife	Y / N	___________
Registered nurse	Y / N	___________
Enrolled nurse	Y / N	___________
Other nurse (LIST):	Y / N	___________
Social worker	Y / N	___________
Counselor	Y / N	___________
Lay counselor	Y / N	___________
Pharmacist	Y / N	___________
Pharmacy assistant	Y / N	___________
Other (LIST):	Y / N	___________
Other (LIST):	Y / N	___________
Other (LIST):	Y / N	___________

Today's Date					
d	d	m	m	y	r

Participant ID				
S				

7. a. Has your staffing changed significantly in the past 5 years?<br><br>b. Why is that?	☐ Yes (1)<br>☐ No (0)<br><br>OPEN RESPONSE:
8. a. IF SERVICE IS NOT HIV OR TB: Do the staff in your department or service have training in HIV and/or TB testing and treatment?<br><br>b. Why is that?	☐ Yes (1)<br>☐ No (0)<br>☐ N/A (88)<br><br>OPEN RESPONSE:
9. a. IF SERVICE IS HIV OR TB: Do the staff in your department or service have training in providing reproductive health services like family planning, antenatal care, TOP or screening for reproductive cancers (e.g. Pap smears, mammograms, etc)?<br><br>b. Why is that?	☐ Yes (1)<br>☐ No (0)<br>☐ N/A (88)<br><br>OPEN RESPONSE:
10. a. IF SERVICE IS HIV OR TB: Do the staff in your department or service have training in providing services for non-communicable diseases like cardiovascular problems, diabetes, respiratory conditions, etc?<br><br>b. Why is that?	☐ Yes (1)<br>☐ No (0)<br>☐ N/A (88)<br><br>OPEN RESPONSE:

Today's Date					
d	d	m	m	y	r

Participant ID				
S				

SECTION 3: SERVICES

INTRODUCTION: I'm interested to know what services are provided by this facility or in this service/department, how patients get services, and referrals to other facilities.

11. As a reminder, because of your role here, the following questions will be for the:<br><br>IF S/HE IS RESPONSIBLE FOR MORE THAN ONE SERVICE, USE EXTRA COPIES OF THIS PAGE, AND ASK SEPARATELY FOR EACH SERVICE.	INDICATE RESPONSE GIVEN IN # 5.<br><br>SERVICE: ________________________

Table 2 (NB: Tx= treatment, Sc = screening, mgmt. = management)

A. Service	B. Days of the week available	C. Hours available	D. If not offered, where are patients referred to for services?
**HIV / AIDS:**			
Testing & Counselling			
ART Initiation			
Routine Treatment (Adults)			
Routine Treatment (Children)			
PMTCT			
Other			
**Tuberculosis:**			
Testing/Diagnosing			
Treatment/Routine Care			
Other			
**Women's Health:**			
TOP			
Cervical cancer screening			
Cervical cancer treatment			
Breast cancer screening			
Breast cancer treatment			
OB / GYN			
Other			
**Reproductive Health:**			
Family planning			
Sexually Transmitted Infections			
Other			
**Chronic Disease Mgmt:**			
Obesity			

Today's Date					
d	d	m	m	y	r

Participant ID				
**S**				

Diabetes			
Hypertension			
Respiratory Disease			
Cardiovascular Disease			
Other			
**Immunisations / Childhood Health:**			
**Mental Health:**			
**Prevention:**			
Well Child			
Medical Male Circumcision			
Other			
**Other:**			

Today's Date					
d	d	m	m	y	r

Participant ID				
S				

12. a. In this study we're interested in "integration of services"Integration can be done in many ways. For example, it can mean one health care provider offering many different services in the same visit, or one clinic offering many services on the same day. Do you think integration of HIV into other primary health care services is a good idea?	☐ No (0)<br>☐ Yes (1)<br>☐ It depends... (3)<br>☐ Unsure (4)
b. Why do you think that?	OPEN RESPONSE:
13. a. Have there been any efforts to integrate HIV testing and treatment into the services at this clinic?	☐ No (0)<br>☐ Yes (1)
b. IF YES, can you describe those? [PROBE: When? Why? Was it successful?]	OPEN RESPONSE:
c. IF NO, can you say why you think that is?	OPEN RESPONSE:

Today's Date					
d	d	m	m	y	r

Participant ID				
S				

14. a. As I noted above, integration can be done in many ways. Do you think integration of TB into other primary health care services is a good idea?	☐ No (0)<br>☐ Yes (1)<br>☐ It depends... (3)<br>☐ Unsure (4)
b. Why do you think that?	OPEN RESPONSE:
15. a. Have there been any efforts to integrate TB testing and treatment into other services at the primary health clinics in the district?	☐ No (0)<br>☐ Yes (1)
b. IF YES, can you describe those? [PROBE: When? Why? Was it successful?]	OPEN RESPONSE:
c. IF NO, can you say why you think that is?	OPEN RESPONSE:

Today's Date					
d	d	m	m	y	r

Participant ID				
S				

SECTION 4: PATIENTS SERVED AND THEIR HEALTH CARE NEEDS

INTRODUCTION: To finish the interview, I have a few more questions about the clients or patients that you see in the services here.

16. As a reminder, because of your role here, the following questions will be for the:<br><br>IF S/HE IS RESPONSIBLE FOR MORE THAN ONE SERVICE, USE EXTRA COPIES OF THIS PAGES FOR THIS SECTION, AND ASK SEPARATELY FOR EACH SERVICE.	INDICATE RESPONSE GIVEN IN # 5.<br><br>SERVICE: _________________________
17. a. Do you have a system for tracking how many patients you see in this service/department each month?<br><br>b. IF NO, Why is that?<br><br>b. IF YES, Can you describe that system for me?	☐ No (0)<br>☐ Yes (1)<br><br>OPEN RESPONSE<br><br>OPEN RESPONSE
18. Can you tell me on average on many patients you see in this service/department each month? [IF POSSIBLE OBTAIN STATS FOR THE LAST THREE MONTHS.]	Number: ____________ last month<br><br>Number: ____________ month before last<br><br>Number: ____________ three months ago<br><br>Average per month: _____________

Today's Date					
d	d	m	m	y	r

Participant ID				
S				

19. a. IF SERVICE IS NOT HIV OR TB: Do you think that some of the patients that you see in this service also need services for HIV or TB?	☐ Yes – both HIV and TB (1)<br>☐ Yes – HIV only (2)<br>☐ Yes – TB only (3)<br>☐ No (0)<br>☐ Don't know (77)
b. Why is that?	OPEN RESPONSE:
20. a. IF SERVICE IS HIV OR TB: Do you think that some of the patients that you see in this service also need services for other issues – like sexual and reproductive health services or non-communicable diseases?	☐ Yes (1)<br>☐ No (0)<br>☐ Don't know (77)
b. IF NO, Why is that?	OPEN RESPONSE:
c. IF YES, What services do you think they need?	OPEN RESPONSE:
i. Women's Health	i. ☐ No (0) / ☐ Yes (1)
ii. Reproductive Health	ii. ☐ No (0) / ☐ Yes (1)
iii. Chronic Disease Mgmnt	iii. ☐ No (0) / ☐ Yes (1)
iv. Immunisation/Childhood Health	iv. ☐ No (0) / ☐ Yes (1)
v. Mental Health	v. ☐ No (0) / ☐ Yes (1)
vi. Prevention	vi. ☐ No (0) / ☐ Yes (1)
vii. Other	vii. ☐ No (0) / ☐ Yes (1)

Today's Date					
d	d	m	m	y	r

Participant ID				
S				

Interviewer initials and date____________________

Interviewer comments:

