# Supplementary material for: Voices from the front lines: A qualitative study of integration of HIV, tuberculosis, and primary healthcare services in Johannesburg, South Africa
Source: PLoS One. 2020 Oct 8;15(10):e0230849. doi: 10.1371/journal.pone.0230849 (PMC7544124; doi:10.1371/journal.pone.0230849)
Supplement: Attachment — Submitted filename: Response to reviewers.docx [file pone.0230849.s002.docx]

**Response to reviewers**

Comments received from the editor and two reviewers are listed below. We have numbered them for ease in cross-referencing. Responses follow each comment in **bold** text. Line numbers refer to the tracked manuscript.

# Editorial requests

When submitting your revision, we need you to address these additional requirements.
 
Please ensure that your manuscript meets PLOS ONE's style requirements, including those for file naming. The PLOS ONE style templates can be found at
<http://www.journals.plos.org/plosone/s/file?id=wjVg/PLOSOne_formatting_sample_main_body.pdf> and <http://www.journals.plos.org/plosone/s/file?id=ba62/PLOSOne_formatting_sample_title_authors_affiliations.pdf>

1. Please include a copy of the interview guide used in the study, in both the original language and English, as Supporting Information, or include a citation if it has been published previously.

**We used three different interview guides. We have combined them into one pdf and attached.**

2. Thank you for including your financial disclosure statement; "This work was funded by the generous support of the American people through the United States Agency for International Development (USAID), award number AID-674-A-12-00029. The contents are the responsibility of the Health Economics and Epidemiology Research Office, a Division of the Wits Health Consortium (Pty) Ltd and do not necessarily reflect the views of USAID or the United States Government. " We note that you received funding from a commercial source: Wits Health Consortium (Pty) Ltd. Please provide an amended Competing Interests Statement that explicitly states this commercial funder, along with any other [relevant declarations](http://www.plosone.org/static/competing.action) relating to employment, consultancy, patents, products in development, marketed products, etc. Within this Competing Interests Statement, please confirm that this does not alter your adherence to all PLOS ONE policies on sharing data and materials by including the following statement: "This does not alter our adherence to PLOS ONE policies on sharing data and materials.” (as detailed online in our guide for authors <http://journals.plos.org/plosone/s/competing-interests>).  If there are restrictions on sharing of data and/or materials, please state these. Please note that we cannot proceed with consideration of your article until this information has been declared.
Please include your amended Competing Interests Statement within your cover letter. We will change the online submission form on your behalf. Please know it is PLOS ONE policy for corresponding authors to declare, on behalf of all authors, all potential competing interests for the purposes of transparency. PLOS defines a competing interest as anything that interferes with, or could reasonably be perceived as interfering with, the full and objective presentation, peer review, editorial decision-making, or publication of research or non-research articles submitted to one of the journals. Competing interests can be financial or non-financial, professional, or personal. Competing interests can arise in relationship to an organization or another person. Please follow this link to our website for more details on competing interests: <http://journals.plos.org/plosone/s/competing-interests>

**The Wits Health Consortium, (Pty) Ltd is a division of the University of the Witwatersrand. It was established as a private, not-for-profit entity with the intention of providing the controls required to receive funding from groups such as USAID. The Health Economics and Epidemiology Research Office is a division within the Wits Health Consortium. Thus, it is the place of employment for the authors. It is not a funding source.**

3. We note that you have indicated that data from this study are available upon request. PLOS only allows data to be available upon request if there are legal or ethical restrictions on sharing data publicly. For information on unacceptable data access restrictions, please see <http://journals.plos.org/plosone/s/data-availability#loc-unacceptable-data-access-restrictions>.
 
In your revised cover letter, please address the following prompts:

a) If there are ethical or legal restrictions on sharing a de-identified data set, please explain them in detail (e.g., data contain potentially identifying or sensitive patient information) and who has imposed them (e.g., an ethics committee). Please also provide contact information for a data access committee, ethics committee, or other institutional body to which data requests may be sent.

b) If there are no restrictions, please upload the minimal anonymized data set necessary to replicate your study findings as either Supporting Information files or to a stable, public repository and provide us with the relevant URLs, DOIs, or accession numbers. Please see <http://www.bmj.com/content/340/bmj.c181.long> for guidelines on how to de-identify and prepare clinical data for publication. For a list of acceptable repositories, please see <http://journals.plos.org/plosone/s/data-availability#loc-recommended-repositories>.

We will update your Data Availability statement on your behalf to reflect the information you provide.
 
**We have provided the required information in our cover letter.**

# Reviewers' comments

Reviewer's Responses to Questions

**Comments to the Author**

1. Is the manuscript technically sound, and do the data support the conclusions?

The manuscript must describe a technically sound piece of scientific research with data that supports the conclusions. Experiments must have been conducted rigorously, with appropriate controls, replication, and sample sizes. The conclusions must be drawn appropriately based on the data presented. 

Reviewer #2: Partly

Reviewer #3: Partly

**See responses to reviewer comments.**

2. Has the statistical analysis been performed appropriately and rigorously? 

Reviewer #2: Yes

Reviewer #3: N/A

3. Have the authors made all data underlying the findings in their manuscript fully available?

The [PLOS Data policy](http://www.plosone.org/static/policies.action#sharing) requires authors to make all data underlying the findings described in their manuscript fully available without restriction, with rare exception (please refer to the Data Availability Statement in the manuscript PDF file). The data should be provided as part of the manuscript or its supporting information, or deposited to a public repository. For example, in addition to summary statistics, the data points behind means, medians and variance measures should be available. If there are restrictions on publicly sharing data—e.g. participant privacy or use of data from a third party—those must be specified.

Reviewer #2: No

Reviewer #3: No

**See response to data item above – and revised cover letter.**

4. Is the manuscript presented in an intelligible fashion and written in standard English?

PLOS ONE does not copyedit accepted manuscripts, so the language in submitted articles must be clear, correct, and unambiguous. Any typographical or grammatical errors should be corrected at revision, so please note any specific errors here.

Reviewer #2: Yes

Reviewer #3: Yes

5. Review Comments to the Author

Please use the space provided to explain your answers to the questions above. You may also include additional comments for the author, including concerns about dual publication, research ethics, or publication ethics. (Please upload your review as an attachment if it exceeds 20,000 characters)

**See individual responses above and below – in bold.**

# Reviewer #2

This is an interesting paper addressing a current topical issue on the need to integrate health services for HIV and other chronic illness. These are my major comments the authors need to consider in their revision.

1. In the introduction, they give a good overview of the historical process of attempts to integrate HIV services with other diseases or activities. What's missing is a clear definition of what the authors perceive as integration i.e. is it about comprehensive services or some select activities? Within South Africa are there meso-macro level issues to consider e.g. national policies and their implementation e.g. integrated chronic disease model and primary health care re-engineering? It would be good if the authors refer to literature beyond South Africa, as there have been recent evidence on the state of integrating HIV care with other conditions.

**Thanks for this comment. We intentionally left out our “own definition” of integration when approaching the study because we were keen to allow the respondents to define integration themselves. That said, in the Introduction, we do provide a summary of the global definitions (lines 66-69), and we have added a paragraph with more detail on South Africa specifically (lines 70-78). In that paragraph, we try to be more precise with regard to the policies and definitions at play in the country.**

Methods

2. The quantitative data collection has been narrowly described. There is need to provide more information on rationale, data collected and from whom.

**The interview guides have now been added as supplementary material. As noted in the Methods section, the guides were semi-structured, meaning they included both quantitative and open-ended qualitative sections. Thus, the detailed description provided for data collection applies to both the qualitative and quantitative data. We tried to clarify this fact in lines 130-131.**

3. Setting - Any additional evidence on the burden of HIV/TB in the setting of interest (Johannesburg)

**Unfortunately, extremely local data as requested is not available.**

4. The results section is well written with interesting qualitative data. However, some of the quotes are too lengthy and would have liked the authors to engage more on perspectives around how integration can be promoted (beyond HIV/TB), as there are patients with other comorbidities or chronic diseases.

**Thanks for this insight. We have shortened some of the quotes. However, we have not added discussion of chronic diseases to the results as that was outside the scope of the study – i.e. not discussed with the respondents.**

5. The discussion section is too narrow, given the topic being discussed. The authors need to engage more with comparisons with lessons or evidence from other setting, and recent evidence on leveraging HIV care platforms for other chronic diseases. Second, their own reflection of how they perceive integration is lacking or left unaddressed. That is moving forward, what is being promoted, with many disease-specific programmes and a growing population of chronic non-communicable diseases. These need to be addressed.

**We have added a discussion of other data on integration of HIV, TB and sexual and reproductive health services to the Discussion. However, we have not included a summary of general progress in the global dialogue on integration (including on integration with NCDs) as we felt that that was somewhat outside the scope of this study which focused specifically on HIV and TB services in primary healthcare settings.**

6. The study limitations have not been discussed e.g. transferability/generalizability of findings, study design limitation and how these were addressed.

   Minor revisions

**We addressed this comment in the Discussion where we had previously mentioned the study’s limitations (lines 520-537).**

7. Line 121 - 126, page 6. Move the text to new heading on ethical consideration.

**The ethics information is entered into a separate space in the PLOS One submission system, and I believe gets labeled as such in the final product. Thus we have left out a separate heading as requested.**

# Reviewer #3

Summary

1. The study explored opinions and experiences of service managers and health care providers regarding integration of HIV and TB services into primary healthcare services. Respondents were able to identify benefits and barriers to integrated HIV and TB services. Concerns about the effect of integration on waiting times (patients), staff workloads and TB infection control issues were raised.
   Overall impression – the study is worth being investigated following the high infection rates of HIV and TB despite numerous campaigns worldwide. There are some minor adjustments to be made indicated below. The title might need revision, may be delete “qualitative exploration”. Tables are clearly presented and labelled correctly. The discussion section need to be supported by previous studies done, references must be added so that conclusion drawn can also be linked contextually with implications.

**We have updated the title to say, “qualitative study” rather than “qualitative exploration.” Regarding the Discussion section, we have added references to/summaries of other research on integration in South Africa and the region.**

Areas of improvement
Minor revision

2. Introduction - In P5 -The authors aim to explore opinion and experiences regarding integration of HIV and TB services into primary health care services - to achieve this there are several questions highlighted by authors - where and how integration is taking place, why it happened (this can be answered through literature), what it looks like now, possible limitations to its implementation and impact, the role of managers and healthcare providers during and after the transition to integrated care.
   There is a need to integrate the introduction in order to ensure that the questions and the context is clear. Authors may consider adding relevant information and add sources especially with regard to the studies already done in South Africa, some arguments lack depth and some ideas and arguments are unclear lacking clarification with relevant sources (studies done on the same topic).

**We have added a paragraph with more detail on integration in South Africa specifically (lines 70-78). In that paragraph, we try to be more precise with regard to the policies and definitions at play in the country. With regard to “studies already done,” we have added a summary of other research (with citations) to the Discussion as we felt it was more appropriate there.**

Methods and Material

3. Major revision- It might also be worth indicating how Grounded theory was utilized from data collection – since analysis followed grounded theory. Further clarification is critical.

**Grounded theory is a conceptual framework that guides qualitative analysis. Data collection follows other conventions – in our case, for semi-structured interviewing. Our data collection processes are explained, and we have added an explanation and references on grounded theory in lines 155-157.**

Minor revision

4. Sample technique - P5 Out of seven administrative regions - purposive sampling was used but there is no information regarding why the 1 region was chosen, or the three primary health clinics, justification is important.

**We added this justification/explanation in lines 112-114.**

5. P6 – line 114 the question about the historical and ongoing partnership with NGO’s for provision of services with the city was not explored.

**In fact, this is discussed throughout the results, but we realize that it may not be clear. The CCMTs were supported by local NGOs. We have noted that in line 253, so that the reader will know that any reference to a CCMT is a reference to local NGO support. (This is also explained in the Introduction.)**

6. Ethics - P6 line 122 - Authors must indicate the ethical approval reference number from the University of Witwatersrand

**We added the protocol number as assigned by the University.**

Results
Minor revision

7. The results presented are sufficient and relevant.

**Thank you.**

8. P12 – generalizing and answering for 10 clinics might not represent the 7 other clinics without staff from the 7 clinics – given that information from regional/city managers at times differ from the staff who are directly involved.

**We addressed generalizability in the Discussion where we had previously mentioned the study’s limitations (lines 520-537).**

9. P14 – line 293 seem to answer why integration of HIV related services occurred.

**Yes. There are many reasons why integration occurred – elucidating those was one of the aims of this study. We have discussed the reasons – from the respondents’ perspective – throughout the results.**

10. P17 – there is repetition regarding the integration of services being a good idea (line 362, 367; line 370,377), authors might consider report for both regional/city managers and health providers simultaneously to avoid repeating the same information.

**Thanks for this; however, we’re not sure that what is noted here is repetition. The first line – (previously line 362) says that indeed the respondents felt that integration was a good idea. Then the subsequent lines unpack that – offering the reasons why they thought it was a good idea. The reasons are varied – not repeated.**

Discussion
Minor revision

11. The discussion section requires revision in order to add relevant studies and align conclusions with implications. Compare and contrast key findings with other literature and flesh out interpretation of the key findings.

**We have added this information to the Discussion.**

12. Additional comments: The study is significant and contribute knowledge especially regarding the integration of HIV and TB healthcare services.

**Thank you!**

6. If you would like your identity to be revealed to the authors, please include your name here (optional).

Your name and review will not be published with the manuscript. 

Reviewer #2: No

Reviewer #3: (No Response)


[NOTE: If reviewer comments were submitted as an attachment file, they will be attached to this email and accessible via the submission site. Please log into your account, locate the manuscript record, and check for the action link "View Attachments". If this link does not appear, there are no attachment files to be viewed.]


While revising your submission, please upload your figure files to the Preflight Analysis and Conversion Engine (PACE) digital diagnostic tool, <http://pacev2.apexcovantage.com/>. PACE helps ensure that figures meet PLOS requirements. To use PACE, you must first register as a user. Registration is free. Then, login and navigate to the UPLOAD tab, where you will find detailed instructions on how to use the tool. If you encounter any issues or have any questions when using PACE, please email us at [figures@plos.org](mailto:figures@plos.org). Please note that Supporting Information files do not need this step.
