# Supplementary material for: Voices from the front lines: A qualitative study of integration of HIV, tuberculosis, and primary healthcare services in Johannesburg, South Africa
Source: PLoS One. 2020 Oct 8;15(10):e0230849. doi: 10.1371/journal.pone.0230849 (PMC7544124; doi:10.1371/journal.pone.0230849)
Supplement: Attachment — Submitted filename: Response to reviewers (1).docx [file pone.0230849.s003.docx]

**PONE-D-19-17035
Voices from the front lines: A qualitative study of integration of HIV, tuberculosis, and primary healthcare services in Johannesburg, South Africa**

**Response to reviewers**

Comments received from the editor and two reviewers are listed below. We have numbered them for ease in cross-referencing. Note that the lines numbers represent the track-changed version.

Responses follow each comment in **bold** text.

If you would like to make changes to your financial disclosure, please include your updated statement in your cover letter.


Journal Requirements:

When submitting your revision, we need you to address these additional requirements.

1. Please ensure that your manuscript meets PLOS ONE's style requirements, including those for file naming. The PLOS ONE style templates can be found at <http://www.journals.plos.org/plosone/s/file?id=wjVg/PLOSOne_formatting_sample_main_body.pdf> and <http://www.journals.plos.org/plosone/s/file?id=ba62/PLOSOne_formatting_sample_title_authors_affiliations.pdf>

**Done.**

2. We note that you have indicated that data from this study are available upon request. PLOS only allows data to be available upon request if there are legal or ethical restrictions on sharing data publicly. For more information on unacceptable data access restrictions, please see <http://journals.plos.org/plosone/s/data-availability#loc-unacceptable-data-access-restrictions>.

In your revised cover letter, please address the following prompts:

a) If there are ethical or legal restrictions on sharing a de-identified data set, please explain them in detail (e.g., data contain potentially sensitive information, data are owned by a third-party organization, etc.) and who has imposed them (e.g., an ethics committee). Please also provide contact information for a data access committee, ethics committee, or other institutional body to which data requests may be sent.
b) If there are no restrictions, please upload the minimal anonymized data set necessary to replicate your study findings as either Supporting Information files or to a stable, public repository and provide us with the relevant URLs, DOIs, or accession numbers. For a list of acceptable repositories, please see <http://journals.plos.org/plosone/s/data-availability#loc-recommended-repositories>.

We will update your Data Availability statement on your behalf to reflect the information you provide.

**As noted in communication for this manuscript LAST YEAR: the data are considered restricted by the local ethics committee: the Health Research Ethics Committee (medical) at the University of the Witwatersrand. The study was protocol M140104. Permission to use the data can be obtained through a request to the Committee. Contact details are as follows:**

**Research Office Secretariat**

**Faculty of Health Sciences**

**Phillip Tobias Building, 3^rd^ Floor, Office 301**

**29 Princess of Wales Terrace**

**Parktown 2193**

**Johannesburg, South Africa**

**Telephone: +27 (0)11 717 1252**

[**hrec-medical.researchoffice@wits.ac.za**](mailto:hrec-medical.researchoffice@wits.ac.za)

[**www.wits.ac.za/research/about-our-research/ethics-and-research-integrity/**](http://www.wits.ac.za/research/about-our-research/ethics-and-research-integrity/)

**The information is now provided in the revised cover letter.**

3. Thank you for stating the following in the Financial Disclosure section:

' This study was made possible by the generous support of the American people through the United States Agency for International Development (USAID), award number AID-674-A-12-00029. The contents are the responsibility of the Health Economics and Epidemiology Research Office, a Division of the Wits Health Consortium (Pty) Ltd and do not necessarily reflect the views of USAID or the United States Government.

LL received the grant.'

We note that you received funding from a commercial source: Wits Health Consortium (Pty) Ltd

Please provide an amended Competing Interests Statement that explicitly states this commercial funder, along with any other [relevant declarations](http://www.plosone.org/static/competing.action) relating to employment, consultancy, patents, products in development, marketed products, etc.

Within this Competing Interests Statement, please confirm that this does not alter your adherence to all PLOS ONE policies on sharing data and materials by including the following statement: "This does not alter our adherence to PLOS ONE policies on sharing data and materials.” (as detailed online in our guide for authors <http://journals.plos.org/plosone/s/competing-interests>).  If there are restrictions on sharing of data and/or materials, please state these. Please note that we cannot proceed with consideration of your article until this information has been declared.

Please include your amended Competing Interests Statement within your cover letter. We will change the online submission form on your behalf.

Please know it is PLOS ONE policy for corresponding authors to declare, on behalf of all authors, all potential competing interests for the purposes of transparency. PLOS defines a competing interest as anything that interferes with, or could reasonably be perceived as interfering with, the full and objective presentation, peer review, editorial decision-making, or publication of research or non-research articles submitted to one of the journals. Competing interests can be financial or non-financial, professional, or personal. Competing interests can arise in relationship to an organization or another person. Please follow this link to our website for more details on competing interests: <http://journals.plos.org/plosone/s/competing-interests>

**As noted in communication for this manuscript LAST YEAR: The Wits Health Consortium, (Pty) Ltd is a division of the University of the Witwatersrand. It was established as a private, not-for-profit entity with the intention of providing the controls required to receive funding from groups such as USAID. The Health Economics and Epidemiology Research Office is a division within the Wits Health Consortium. Thus, it is the place of employment for the authors. It is not a funding source.**

Additional Editor Comments (if provided):

[Note: HTML markup is below. Please do not edit.]

Reviewers' comments:

Reviewer's Responses to Questions

**Comments to the Author**

1. Is the manuscript technically sound, and do the data support the conclusions?

The manuscript must describe a technically sound piece of scientific research with data that supports the conclusions. Experiments must have been conducted rigorously, with appropriate controls, replication, and sample sizes. The conclusions must be drawn appropriately based on the data presented.

Reviewer #1: Yes

**Thank you.**

2. Has the statistical analysis been performed appropriately and rigorously?

Reviewer #1: N/A

3. Have the authors made all data underlying the findings in their manuscript fully available?

The [PLOS Data policy](http://www.plosone.org/static/policies.action#sharing) requires authors to make all data underlying the findings described in their manuscript fully available without restriction, with rare exception (please refer to the Data Availability Statement in the manuscript PDF file). The data should be provided as part of the manuscript or its supporting information, or deposited to a public repository. For example, in addition to summary statistics, the data points behind means, medians and variance measures should be available. If there are restrictions on publicly sharing data—e.g. participant privacy or use of data from a third party—those must be specified.

Reviewer #1: No

**See above.**

4. Is the manuscript presented in an intelligible fashion and written in standard English?

PLOS ONE does not copyedit accepted manuscripts, so the language in submitted articles must be clear, correct, and unambiguous. Any typographical or grammatical errors should be corrected at revision, so please note any specific errors here.

Reviewer #1: Yes

**Thank you.**

5. Review Comments to the Author

Please use the space provided to explain your answers to the questions above. You may also include additional comments for the author, including concerns about dual publication, research ethics, or publication ethics. (Please upload your review as an attachment if it exceeds 20,000 characters)

Reviewer #1: This is indeed an interesting paper that adds value to the knowledge and current issues relating to integration of health services for HIV and TB.
Introduction, methods, results and discussion - The current version address all comments made previously.

**Thank you.**

Recommendation - Table 1 assist readers to have a clear understanding of the study population but Table 2 does not really add weight to the paper. It is therefore recommended that authors remove Table 2 and add a paragraph with information that authors perceive useful and critical in the description of the study population.

**We feel that Table 2 provides the reader with an at-a-glace view of the range of SRH, HIV and TB services provided at the facilities and that this is an important aid when reading through the presentation of the challenges, etc. with integration of care. Table 2 also provides a sense of the volume of patients and the number of staff managing them. We feel that this information could not all be converted to text, and that it is important as a reference.**

Overall impression - the study is worth being published given the level of infection in South Africa.

**Thank you.**

6. PLOS authors have the option to publish the peer review history of their article ([what does this mean?](https://journals.plos.org/plosone/s/editorial-and-peer-review-process#loc-peer-review-history)). If published, this will include your full peer review and any attached files.


If you choose “no”, your identity will remain anonymous but your review may still be made public.


**Do you want your identity to be public for this peer review?** For information about this choice, including consent withdrawal, please see our [Privacy Policy](https://www.plos.org/privacy-policy).

Reviewer #1: No


[NOTE: If reviewer comments were submitted as an attachment file, they will be attached to this email and accessible via the submission site. Please log into your account, locate the manuscript record, and check for the action link "View Attachments". If this link does not appear, there are no attachment files to be viewed.]

While revising your submission, please upload your figure files to the Preflight Analysis and Conversion Engine (PACE) digital diagnostic tool, <https://pacev2.apexcovantage.com/>. PACE helps ensure that figures meet PLOS requirements. To use PACE, you must first register as a user. Registration is free. Then, login and navigate to the UPLOAD tab, where you will find detailed instructions on how to use the tool. If you encounter any issues or have any questions when using PACE, please email us at [figures@plos.org](mailto:figures@plos.org). Please note that Supporting Information files do not need this step.

**There are no figures in this manuscript.**
